# Malnutrition and risks of atrial fibrillation recurrence after catheter ablation

**DOI:** 10.1002/joa3.13196

**Published:** 2024-12-01

**Authors:** Phuuwadith Wattanachayakul, Thitiphan Srikulmontri, Vitchapong Prasitsumrit, Thanathip Suenghataiphorn, Pojsakorn Danpanichkul, Jakrin Kewcharoen, Nipith Charoenngam, Sumeet Mainigi

**Affiliations:** ^1^ Department of Medicine Jefferson Einstein Hospital Philadelphia Pennsylvania USA; ^2^ Sidney Kimmel Medical College Thomas Jefferson University Philadelphia Pennsylvania USA; ^3^ Department of Medicine, Faculty of Medicine Siriraj Hospital Mahidol University Bangkok Thailand; ^4^ Department of Medicine Griffin Hospital Derby Connecticut USA; ^5^ Department of Medicine Texas Tech University Lubbock Texas USA; ^6^ Division of Cardiology University of California san Francisco San Francisco California USA; ^7^ Endocrine Unit Massachusetts General Hospital, Harvard Medical School Boston Massachusetts USA; ^8^ Division of Cardiovascular Disease Jefferson Einstein Hospital Philadelphia Pennsylvania USA

**Keywords:** atrial fibrillation, catheter ablation, malnutrition, meta‐analysis, systematic review

## Abstract

**Background:**

Recent data showed an association between malnutrition and increased all‐cause mortality and thromboembolic risk in patients with atrial fibrillation (AF). However, the impact of malnutrition on the clinical outcomes for patients undergoing catheter ablation for AF is still debated. Our study aimed to examine this relationship using all existing available data.

**Methods:**

We conducted a systematic review of MEDLINE and EMBASE databases from inception to April 2024, analyzing the association between malnutrition, assessed by the Geriatric Nutritional Risk Index (GNRI), and the risk of AF recurrence in patients who underwent catheter ablation for AF, compared to those without malnutrition. Relative Risk (RR) or hazard ratio (HR) and 95% CIs were retrieved from each study and combined using the generic inverse variance method.

**Results:**

We included 3 cohort studies with 1697 participants undergoing AF ablation (10.9%) who had malnutrition indicated by GNRI score below 98. Patients with malnutrition had a higher risk of AF recurrence following catheter ablation for AF compared to those without malnutrition (Pooled RR = 2.74, 95% CI 1.36–5.51, *I* ^2^ = 67%, *p* = .005).

**Conclusions:**

Our pooled analysis indicates that malnourished patients undergoing catheter ablation for AF have an increased risk of AF recurrence compared to non‐malnourished patients.

## Graphical abstract

Our meta‐analysis found that patients undergoing AF ablation with malnutrition (GNRI <98) had a significantly higher risk of AF recurrence after the procedure (Pooled RR = 2.74, 95% CI 1.36–5.51, *I* ^2^ = 67%, *p* = .005).

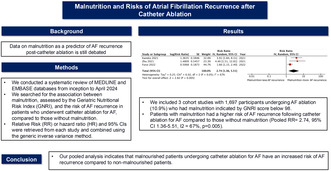

## 1 INTRODUCTION

Atrial fibrillation (AF) is the most common sustained arrhythmia seen in clinical settings and currently affects more than 50 million people globally.^1^, ^2^ The incidence and prevalence of AF continues to rise, with a lifetime risk of about 25% for individuals over the age of 40.^3^ As a significant public health concern, AF significantly increases the risk of heart failure by 5‐fold and stroke by 2.4‐fold while also nearly doubling the risk of mortality.^4^ International guidelines advocated for integrated care for patients with AF, focusing on anticoagulation to prevent strokes, better symptom control, and optimizing cardiovascular risk factors and comorbidities.^5^, ^6^ Catheter ablation has emerged as the cornerstone of rhythm control for AF, surpassing antiarrhythmic drugs (AADs) in efficacy, reducing healthcare utilization, and maintaining comparable safety.^6^, ^7^ Current clinical guideline recommends catheter ablation as the first‐line treatment for rhythm control in selected patients with AF.^6^

Malnutrition, characterized by inadequate nutrition intake resulting in changes to body composition and diminished health function, is becoming more prevalent among patients with cardiovascular disease, significantly increasing mortality rates.^8^, ^9^ Extensive data have shown that malnutrition is a crucial determinant of adverse outcomes in patients with acute decompensated heart failure (ADHF), myocardial infarction, and AF, increasing in‐hospital mortality, extended hospital stays, and numerous systemic complications.^10^, ^11^, ^12^ In light of these findings, current guidelines now prioritize assessing and optimizing nutritional status for patients with cardiovascular conditions, aiming to mitigate the adverse impacts of malnourishment and improve overall patient outcomes.^13^, ^14^

Incidence of AF recurrence following an initial ablation procedure reported to be around 30% to 40%.^15^ Approximately 11% of patients undergoing their first ablation in the United States would require a repeat ablation within 1 year.^16^ Several predictors of AF recurrence following catheter ablation include age, gender, prior chronic medical history, and pre‐ablation echocardiographic findings, all crucial for pre‐procedural planning and ongoing patient monitoring.^17^, ^18^, ^19^, ^20^ Malnutrition has emerged as a potential new risk factors of AF recurrence post‐catheter ablation, however, data remains scarce.^21^, ^22^, ^23^ Thus, we conducted a systematic review and meta‐analysis of observational studies examining the impact of malnutrition on the risk of AF recurrence post‐catheter ablation.

## 2 METHOD

### 2.1 Search strategy

Three investigators (PW, TS, and VP) independently conducted searches in Medline and Embase databases from inception through April 2024 using search terms related to “malnutrition,” “undernutrition,” “nutritional disorder,” “protein deficiency” and combined with the terms “atrial fibrillation,” and “catheter ablation.” No language restrictions were applied. The same investigators independently assessed the eligibility of the retrieved records, with further discussions involving another investigator (AA) to resolve any conflicts. Abstracts and unpublished studies were excluded.

### 2.2 Eligibility criteria

The eligibility criteria were as follows: Included studies must be observational studies (i.e., case–control, cross‐sectional, or cohort) published as original research, assessing the association between pre‐ablation malnutrition and risks of AF recurrence after catheter ablation. Malnutrition is defined as the Geriatric Nutritional Risk Index (GNRI) below 98, Studies must include a group of individuals with malnutrition and another group without malnutrition who underwent catheter ablation for AF and must provide effect estimates representing the association between malnutrition and the risk of AF recurrence in the form of odds ratios (OR), relative risk (RR), or hazard ratios (HR), accompanied by 95% confidence intervals (CIs) or survival curves. Raw data adequate for calculation for the effect sizes is acceptable.

### 2.3 Data extraction

We employed a standardized data collection protocol to extract the following information: last name of the first author, study country, study design, publication year, total number of participants, recruitment protocol, malnutrition diagnosis, types of AF, catheter ablation type, diagnosis of AF recurrence, follow‐up duration, baseline characteristics and comorbidities, mean left ventricular ejection fraction (LVEF), and variables adjusted for in multivariate analysis.

Two investigators (PW and AA) applied the Newcastle‐Ottawa Scale for cohort studies to evaluate research quality, focusing on the quality of participant recruitment, comparability between groups, and accuracy of outcome ascertainment.^24^

### 2.4 Statistical analysis

Data analysis was conducted using Review Manager 5.3 software from the Cochrane Collaboration. Point estimates with standard errors from each study were combined using DerSimonian and Laird's generic inverse variance method.^25^ Due to heterogeneous background populations and protocols among the studies, a random‐effects model was employed. Statistical heterogeneity was assessed using Cochran's *Q* test, supplemented by *I* ^2^ statistics to quantify the proportion of total variation across studies attributable to heterogeneity rather than chance. *I* ^2^ values categorize heterogeneity as insignificant (0%–25%), low (26%–50%), moderate (51%–75%), or high (>75%).^26^ A funnel plot will be utilized to examine potential publication bias if sufficient studies are available.

## 3 RESULTS

**FIGURE 1:**
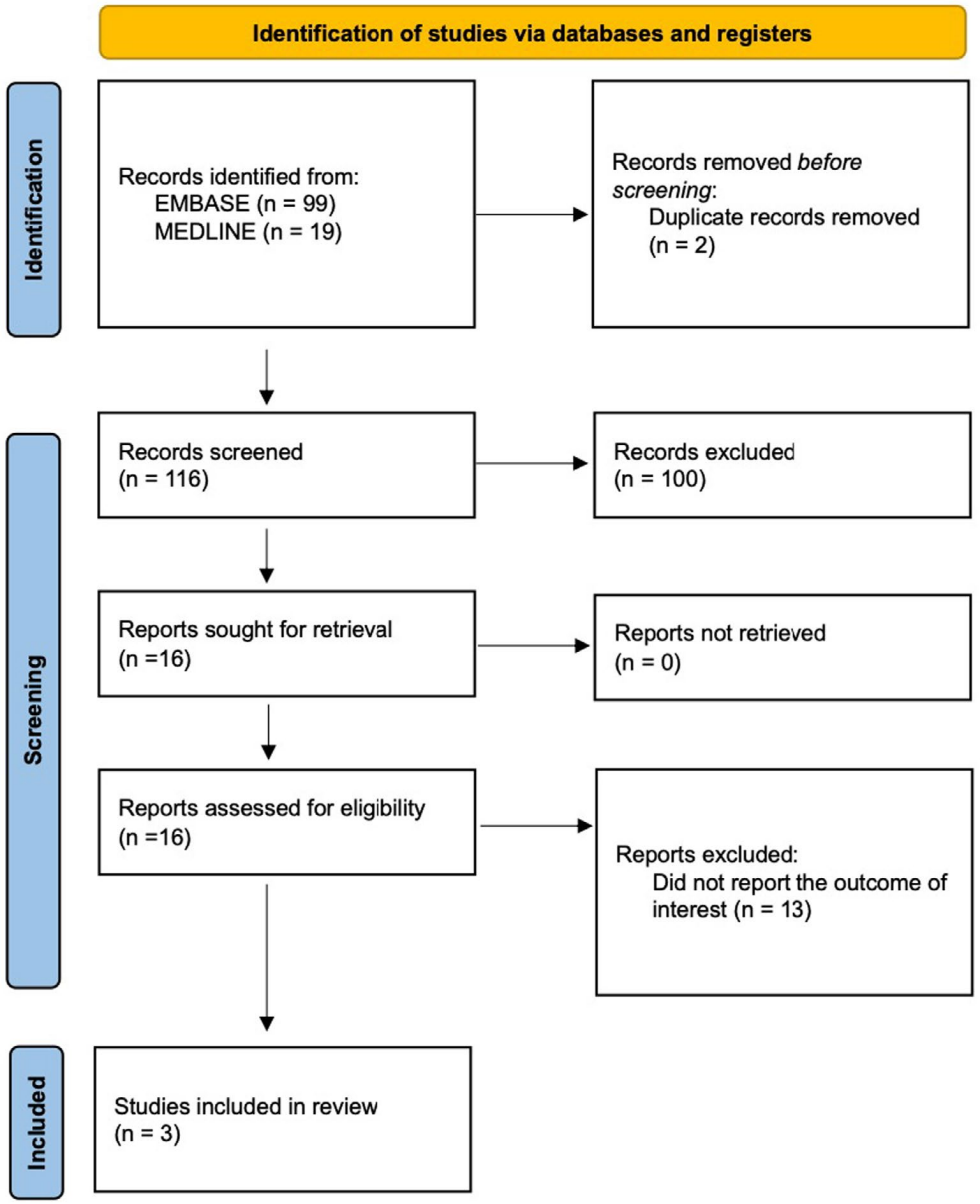
Study identification and literature review process.

Our search strategy identified 118 studies, with 99 from EMBASE and 19 from MEDLINE. After removing two duplicates, we reviewed 116 studies by title and abstract, excluding 100 that did not meet the eligibility criteria related to study design, participants, or article type. Subsequently, we thoroughly reviewed 16 articles and excluded 13 for not reporting the relevant outcome. Ultimately, three studies met the eligibility criteria for our meta‐analysis.^21^, ^22^, ^23^ Figure 1 illustrates our search methodology and selection process, and Table 1 details the characteristics and quality assessment of the included studies.

**TABLE 1:** Main characteristics of the cohort studies included in the meta‐analysis.

	Kaneko et al.^22^	Zhu et al.^23^	Furui et al.^21^
Year of publication	2021	2021	2022
Country of origin	Japan	China	Japan
Study design	Retrospective cohort Study	Retrospective cohort Study	Retrospective cohort Study
Total number of participants (cases)	All participants: 538 Malnutrition: 57 No Malnutrition: 481	All participants: 246 Malnutrition: 51 No malnutrition: 195	All participants: 913 Malnutrition: 77 No malnutrition: 836
Recruitment of participants	Patients underwent their first AF catheter ablation at Japanese Red Cross Musashino Hospital between January 2017 and June 2019 were included in the study.	Patients with non‐valvular, drug‐refractory AF who underwent catheter ablation at Nanfang Hospital between January 2017 and January 2019 were included in the study.	Patients who underwent AF catheter ablation at Ogaki Municipal Hospital in Japan from November 2011 to November 2017 were identified for the study.
Diagnosis of malnutrition	Malnutrition was identified in patients undergoing AF catheter ablation if their pre‐procedure GNRI score was below 98	Malnutrition was identified in patients undergoing AF catheter ablation if their pre‐procedure GNRI score was below 98	Malnutrition was identified in patients undergoing AF catheter ablation if their pre‐procedure GNRI score was below 98
Type of atrial fibrillation	Paroxysmal AF: 68.8% (Malnutrition: 68.2%, No Malnutrition: 70.2%) Persistent AF: 27.9% (Malnutrition: 27.7%, No Malnutrition: 28.1%) Long‐standing Persistent AF: 3.3% (Malnutrition: 4.2%, No Malnutrition: 1.8%)	All participants Paroxysmal AF: 63.8% Persistent AF: 36.2%	Paroxysmal AF: 72.0% (Malnutrition: 56%, No Malnutrition: 56%) Persistent AF: 25.0% (Malnutrition: 27%, No Malnutrition: 25%) Long‐standing Persistent AF: 3.0% (Malnutrition: 17%, No Malnutrition: 19%)
Type of catheter ablation	Radiofrequency or cryoablation	Radiofrequency or cryoablation	Radiofrequency and cryoablation
Diagnosis of atrial fibrillation recurrence	Arrhythmic recurrence was defined as any episode of AF or AT lasting over 30 s, as recorded by Holter ECG or CIEDs/ICMs. A 3‐month blanking period following the ablation was established, during which any arrhythmia recurrences were not counted.	AF recurrence was defined as any atrial arrhythmia lasting more than 30 s, occurring after the initial 3 months, as detected by 12‐lead ECG or 24‐hour Holter monitoring.	AF recurrence was defined as AF episodes lasting more than 30 s, recorded via 12‐lead ECG and Holter monitoring.
Follow‐up duration	Mean: 422 days	Mean: 11.4 ± 7.3 months	Mean: 2.3 ± 0.8 years
Average age of participants (years)	All participants: 67.0 Malnutrition: 75.0 No Malnutrition: 67.0	All participants: 60.6	All participants: 67 Malnutrition: 71.3 No Malnutrition: 66.5
Percentage of male (%)	All participants: 69.9 Malnutrition: 49.1 No Malnutrition: 72.3	All participants: 65.4	All participants: 72.0 Malnutrition: 51.0 No Malnutrition: 74.0
Body mass index (Mean)	Malnutrition: 20.1 ± 1.1 No Malnutrition: 24.3 ± 2.0	All participants: 24.6 ± 3.5	All participants: 23.9 ± 3.6 Malnutrition: 20.1 ± 3.2 No Malnutrition: 24.3 ± 3.4
LVEF (mean)	Malnutrition: 64 No Malnutrition: 66	All participants: 60	Malnutrition: 58.5 No Malnutrition: 63.7
Comorbidities	Malnutrition HTN 50.9% HF 35.1% DM 10.5% Stroke or TIA 7.0% MI 8.8% CKD 52.6% Malignancy 10.5% No Malnutrition HTN 51.4% HF 10.4% DM 12.9% Stroke or TIA 5.4% MI 3.7% CKD 33.3% Malignancy 6.2%	All participants HTN 50% DM: 21.1% CAD: 16.7% Stroke 13.8% Recurrence group HTN: 44.2% DM: 23.4% CAD: 15.6% Stroke 16.9% Non‐recurrence group HTN: 52.7% DM: 20.1% CAD: 17.2% Stroke 12.4%	Malnutrition HTN: 44% DM: 18% HF: 33% Stroke or TIA: 9.1% CAD: 12% No Malnutrition HTN: 61% DM: 19% HF: 18% Stroke or TIA: 8.1% CAD: 8.7%
Variables adjusted in multivariate analysis	Age, gender, heart failure, chronic kidney disease, type of AF, beta blocker usage, pre‐procedural antiarrhythmic drug usage, post‐procedural antiarrhythmic drug usage, diuretic usage, LVEF, LAD, CHADS2 score, CHADS2VASC score, eGFR, BNP, hemoglobin, lymphocyte count, cryoablation, posterior wall isolation, Cavo tricuspid isthmus block line, superior vena cava isolation, GNRI‐based nutritional risk	Age, gender, BMI, type and duration of atrial fibrillation, NYHA class, hypertension, diabetes, coronary heart disease, NT‐pro BNP, LAD, LVEF, antiarrhythmic drugs and early recurrence.	Age, gender, CONUT score, GNRI score, eGFR, LAD, LVEF, PAF, coronary artery disease, and history of HF
Newcastle‐Ottawa score	Selection: 4 Comparability: 1 Outcome: 2	Selection: 4 Comparability: 1 Outcome: 2	Selection: 4 Comparability: 1 Outcome: 2

Abbreviations: AF, Atrial Fibrillation; BMI, Body mass index; BNP, Brain natriuretic peptide; CIED, Cardiovascular Implantable Electronic Devices; CONUT, Controlling Nutritional Status Score; ECG, Electrocardiogram; eGFR, Estimated glomerular filtration rate; GNRI, Geriatric Nutrition Risk Index; HF, Heart Failure; LAD, Left atrium diameter; LVEF, Left ventricular ejection fraction; NYHA, New York Heart Association Functional class.

**FIGURE 2:**
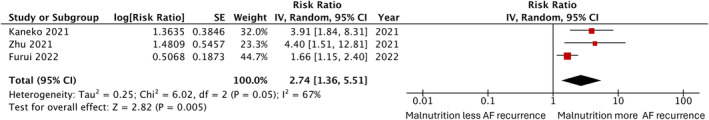
Forest plot of meta‐analysis.

A total of three cohort studies with 1697 participants investigated the association between malnutrition and the recurrence of AF after catheter ablation. The average participant age was 64.8 years old, 69.1% were male, and 68.2% had paroxysmal AF as shown in Table 1. The pooled analysis revealed that individuals with malnutrition had an increased risk of AF recurrence following catheter ablation, with a pooled RR of 2.74 (95% CI: 1.36–5.51, *p* = .005, Figure 2), when compared to individuals without malnutrition. Moderate statistical heterogeneity was observed (*I* ^2^ = 67%). Since only three studies were included in this meta‐analysis, the evaluation for publication bias was not performed.

## 4 DISCUSSION

This study is the first systematic review and meta‐analysis to consolidate data on how malnutrition affects AF recurrence following catheter ablation. The pooled analysis indicated that malnourished patients have a 2.74 times higher risk of AF recurrence following catheter ablation than individuals without malnutrition.

While the exact mechanisms remain uncertain, several potential explanations exist. Malnutrition is closely linked to hyperinflammatory and hypercatabolic states, which hasten the progression of cardiovascular diseases and lead to adverse outcomes in patients with heart failure, myocardial infarction, and conduction system diseases.^27^, ^28^, ^29^ Recent data has identified malnutrition as a potential arrhythmogenic factor, increasing the risk of developing AF and other arrhythmias such as premature atrial contractions and premature ventricular contractions.^30^ The imbalance between pro‐inflammatory and anti‐inflammatory biomarkers is believed to contribute to the recurrence of arrhythmias in malnourished patients.^31^, ^32^, ^33^ For example, elevated pro‐inflammatory biomarkers such as tumor necrosis factor‐alpha (TNF‐α), interleukin‐6 (IL‐6), and C‐reactive protein (CRP) are commonly seen in cases of malnutrition.^34^, ^35^, ^36^ These inflammatory biomarkers function as arrhythmogenic factors, affecting cardiac adipocytes and various immune cells, which in turn disrupt the cell membranes of cardiac cells.^37^ For all these reasons, hyperinflammatory states could damage the cellular and electrical properties of cardiac cells, leading to more frequent recurrent AF post‐catheter ablation. On the other hand, decreased levels of anti‐inflammatory biomarkers, such as myostatin and connexin 40, have been noted in malnourished patients. These proteins play essential roles in regulating the sinoatrial node and electrical signal propagation across the atrial myocardium, suppressing AF development.^38^, ^39^ Therefore, disturbances in these proteins secondary to malnutrition may also contribute to the recurrence of AF following ablation procedures.

Malnutrition is also often associated with sarcopenia (loss of muscle mass and function) and decreased body adipose tissue mass.^40^ These conditions lead to abnormal neurohormonal activation, increasing the risk of myocardial remodeling and electrophysiological changes such as atrial fibrosis, atrophy, and reduced cellular connectivity—key drivers for the recurrence of arrhythmias.^41^ Interestingly, studies have shown that adipose tissue has an anti‐inflammatory activity by expressing TNF‐ α type I and II receptors that bind excess inflammatory cytokines.^42^, ^43^ In malnourished individuals, diminished body fat undermined this ability, reducing overall anti‐arrhythmogenic effects and causing higher AF recurrence.^44^ Finally, malnourished patients often have deficiencies in trace elements and vitamins, contributing to increased AF recurrence after ablation and poor long‐term cardiovascular outcomes.^45^ These interconnected factors highlight the complex relationship between malnutrition and recurrent AF post‐catheter ablation, underscoring the importance of comprehensive nutritional assessments and interventions for these patients.

The findings of this study carry significant clinical implications for managing AF in patients undergoing catheter ablation. The observed link between malnutrition and a higher risk of AF recurrence underscores the importance of integrating comprehensive nutritional assessments into the pre‐procedural evaluation of AF patients. Using tools like the GNRI scoring system can help clinicians identify patients at nutritional risk, allowing for more tailored and holistic management strategies. Moreover, malnutrition may serve as a surrogate marker for patients with lower cardiometabolic reserve, potentially indicating a sicker population at greater risk for poor outcomes following ablation. It's important to note that in all the studies included in this analysis, patients classified as malnourished had an average BMI above 20, suggesting that malnutrition can be present even when BMI is within the normal range. This finding reinforces the need for a more nuanced approach, as relying solely on BMI may overlook patients who are at nutritional risk. Ultimately, this study highlights the need for a multidisciplinary approach, incorporating nutrition specialists in the AF care team to enhance patient outcomes.

The current meta‐analysis has several limitations. Firstly, the included meta‐analysis showed moderate heterogeneity, possibly influenced by differences in baseline characteristics such as types of AF (paroxysmal AF vs. persistent AF), follow‐up duration, and prevalence of malnutrition. Secondly, since all studies were conducted in Asia, the findings may not be generalizable to other populations. Lastly, all 3 of the included study utilized only the GNRI scoring system to evaluate malnutrition, although alternatives method including the Mini Nutritional Assessment (MNA), the Screening Tool for the Control of Nutritional Status (CONUT), and the Prognostic Nutritional Index (PNI) are also available.^22^ Therefore, further research is needed to ascertain whether nutritional status determined by other scoring system is associated with AF recurrence post‐catheter ablation or not. Moreover, identifying the most effective scoring system for assessing malnutrition in this specific patient group is crucial to improve outcome predictions. Additionally, the potential impact of malnutrition on periprocedural complications should be further investigated to offer a more comprehensive understanding of its role in patient outcomes. Lastly, while this analysis primarily focused on malnutrition as a key risk factor for AF recurrence, other established factors—such as age, gender, hypertension, diabetes, and atrial size—also play significant roles in recurrence risk. Future studies should incorporate a broader range of variables to better contextualize the impact of malnutrition in relation to these other factors, providing a more comprehensive understanding of patient risk profiles.

## 5 CONCLUSION

Our meta‐analysis found that AF patients identified as malnourished by the GNRI scoring system had a significantly higher risk of recurrence after catheter ablation. However, since the findings are primarily derived from studies in the East Asia region, further research across diverse populations is needed to validate these results and enhance their generalizability.

## AUTHOR CONTRIBUTIONS

All authors had access to the data and a role in writing the manuscript.

## FUNDING INFORMATION

None.

## CONFLICT OF INTEREST STATEMENT

All the authors declare no conflicts of interest.

## ETHICS STATEMENT

For this type of study, Ethics approval is not required.

## PATIENT CONSENT STATEMENT

For this type of study, formal consent is not required.

## Data Availability

The data from the findings of this study were inferred can be obtained from the author upon reasonable request. All data and materials support the published claims and comply with field standards.
